# Ethanaminium 3,4,5,6-tetra­bromo-2-(meth­oxy­carbon­yl)benzoate methanol monosolvate

**DOI:** 10.1107/S1600536810052591

**Published:** 2010-12-18

**Authors:** Jian Li

**Affiliations:** a Department of Chemistry and Chemical Engineering, Weifang University, Weifang 261061, People’s Republic of China

## Abstract

In the crystal structure of the title compound, C_2_H_8_N^+^·C_9_H_3_Br_4_O_4_^−^·CH_4_O, inter­molecular N—H⋯O and O—H⋯O hydrogen bonds link the components into chains along [001]. Additional stabilization is supplied by weak C—H⋯O and C—H⋯Br inter­actions.

## Related literature

For a related structure, see: Liang (2008 ▶).
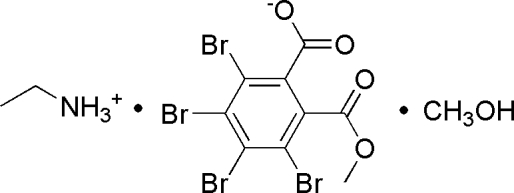


## Experimental

### 

#### Crystal data

C_2_H_8_N^+^·C_9_H_3_Br_4_O_4_^−^·CH_4_O
*M* *_r_* = 572.89
Monoclinic, 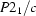

*a* = 9.4651 (8) Å
*b* = 25.6544 (19) Å
*c* = 8.3365 (6) Å
β = 112.787 (1)°
*V* = 1866.3 (2) Å^3^
*Z* = 4
Mo *K*α radiation
μ = 8.64 mm^−1^
*T* = 298 K
0.40 × 0.35 × 0.33 mm

#### Data collection

Bruker SMART CCD diffractometer
Absorption correction: multi-scan (*SADABS*; Bruker, 1997 ▶) *T*_min_ = 0.130, *T*_max_ = 0.163
9436 measured reflections
3295 independent reflections
1454 reflections with *I* > 2σ(*I*)
*R*_int_ = 0.086

#### Refinement

*R*[*F* ^2^ > 2σ(*F* ^2^)] = 0.042
*wR*(*F* ^2^) = 0.068
*S* = 0.99
3295 reflections
200 parameters
H-atom parameters constrained
Δρ_max_ = 0.49 e Å^−3^
Δρ_min_ = −0.51 e Å^−3^

### 

Data collection: *SMART* (Bruker, 1997 ▶); cell refinement: *SAINT* (Bruker, 1997 ▶); data reduction: *SAINT*; program(s) used to solve structure: *SHELXS97* (Sheldrick, 2008 ▶); program(s) used to refine structure: *SHELXL97* (Sheldrick, 2008 ▶); molecular graphics: *SHELXTL* (Sheldrick, 2008 ▶) and *PLATON* (Spek, 2009 ▶); software used to prepare material for publication: *SHELXTL*.

## Supplementary Material

Crystal structure: contains datablocks global, I. DOI: 10.1107/S1600536810052591/lh5189sup1.cif

Structure factors: contains datablocks I. DOI: 10.1107/S1600536810052591/lh5189Isup2.hkl

Additional supplementary materials:  crystallographic information; 3D view; checkCIF report

## Figures and Tables

**Table 1:** Hydrogen-bond geometry (Å, °)

*D*—H⋯*A*	*D*—H	H⋯*A*	*D*⋯*A*	*D*—H⋯*A*
N1—H1*A*⋯O4^i^	0.89	1.94	2.772 (9)	155
N1—H1*B*⋯O5	0.89	1.92	2.810 (9)	174
N1—H1*C*⋯O3^ii^	0.89	1.99	2.871 (7)	170
N1—H1*C*⋯O4^ii^	0.89	2.58	3.249 (8)	132
O5—H5⋯O3	0.82	1.90	2.705 (8)	165
C10—H10*A*⋯Br3^iii^	0.97	2.92	3.736 (8)	143
C11—H11*B*⋯O2^iv^	0.96	2.47	3.357 (10)	153

Symmetry codes: (i) 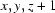; (ii) 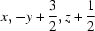; (iii) 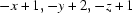; (iv) 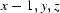.

**Fig. 1.**
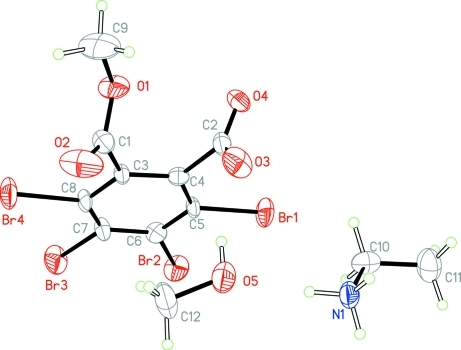
The asymmetric unit of (I), drawn with 30% probability ellipsoids.

**Fig. 2.**
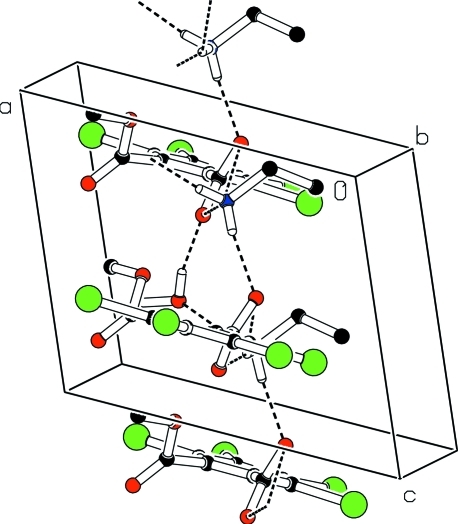
The crystal packing of (I). Hydrogen bonds are indicated by dashed lines.

## supplementary crystallographic information

### Comment

4,5,6,7-Tetrabromo-2-ethylisoindoline-1,3-dione is an important flame retardant. 2-(Methoxycarbonyl)-3,4,5,6-tetrabromobenzoic acid is an intermediate in the sytnthesis of this flame retardant. In this paper, the structure of the title compound is reported. The asymmetric unit of the title compound (I) contains one ethanaminium cation, one 2-(methoxycarbonyl)-3,4,5,6-tetrabromobenzoate anion and one methanol solvent molecule (Fig. 1). The bond lengths and angles agree with those in ethane-1,2-diaminium 2-(methoxycarbonyl)-3,4,5,6-tetrabromo benzoate methanol solvate (Liang, 2008). In the crystal structure, intermolecular N—H···O and O—H···O hydrogen bonds link the components of the structure into one-dimensional chains along [001](see fig. 2). Additional stabilization is supplied by weak C—H···O and C—H···Br interactions.

### Experimental

A mixture of 4,5,6,7-tetrabromoisobenzofuran-1,3-dione (4.64 g, 0.01 mol) and methanol (15 ml) was refluxed for 0.5 h. And then ethanamine (0.45 g, 0.01 mol) was added to the above solution, and mixed for 10 min at room temperature. The solution was kept at room temperature for 3 d. Natural evaporation gave colourless single crystals of the title compound, suitable for X-ray analysis.

### Refinement

H atoms were initially located from difference maps and then refined in a riding model with C—H = 0.96–0.97 Å, N—H = 0.89 Å, O-H = 0.82Å and *U*_iso_(H) = 1.2*U*_eq_(C) or 1.5*U*_eq_(O, N, methyl C).

### Crystal data

**Table:**

C_2_H_8_N^+^·C_9_H_3_Br_4_O_4_^−^·CH_4_O	*F*(000) = 1096
*M**_r_* = 572.89	*D*_x_ = 2.039 Mg m^−^^3^
Monoclinic, *P*2_1_/*c*	Mo *K*α radiation, λ = 0.71073 Å
Hall symbol: -P 2ybc	Cell parameters from 1513 reflections
*a* = 9.4651 (8) Å	θ = 2.3–20.6°
*b* = 25.6544 (19) Å	µ = 8.64 mm^−^^1^
*c* = 8.3365 (6) Å	*T* = 298 K
β = 112.787 (1)°	Block, colorless
*V* = 1866.3 (2) Å^3^	0.40 × 0.35 × 0.33 mm
*Z* = 4	

### Data collection

**Table:**

Bruker SMART CCD diffractometer	3295 independent reflections
Radiation source: fine-focus sealed tube	1454 reflections with *I* > 2σ(*I*)
graphite	*R*_int_ = 0.086
φ and ω scans	θ_max_ = 25.0°, θ_min_ = 2.3°
Absorption correction: multi-scan (*SADABS*; Bruker, 1997)	*h* = −9→11
*T*_min_ = 0.130, *T*_max_ = 0.163	*k* = −30→30
9436 measured reflections	*l* = −9→8

### Refinement

**Table:**

Refinement on *F*^2^	Primary atom site location: structure-invariant direct methods
Least-squares matrix: full	Secondary atom site location: difference Fourier map
*R*[*F*^2^ > 2σ(*F*^2^)] = 0.042	Hydrogen site location: inferred from neighbouring sites
*wR*(*F*^2^) = 0.068	H-atom parameters constrained
*S* = 0.99	*w* = 1/[σ^2^(*F*_o_^2^) + (0.0024*P*)^2^] where *P* = (*F*_o_^2^ + 2*F*_c_^2^)/3
3295 reflections	(Δ/σ)_max_ = 0.001
200 parameters	Δρ_max_ = 0.49 e Å^−^^3^
0 restraints	Δρ_min_ = −0.51 e Å^−^^3^

### Special details

**Table:**

Geometry. All e.s.d.'s (except the e.s.d. in the dihedral angle between two l.s. planes) are estimated using the full covariance matrix. The cell e.s.d.'s are taken into account individually in the estimation of e.s.d.'s in distances, angles and torsion angles; correlations between e.s.d.'s in cell parameters are only used when they are defined by crystal symmetry. An approximate (isotropic) treatment of cell e.s.d.'s is used for estimating e.s.d.'s involving l.s. planes.
Refinement. Refinement of *F*^2^ against ALL reflections. The weighted *R*-factor *wR* and goodness of fit *S* are based on *F*^2^, conventional *R*-factors *R* are based on *F*, with *F* set to zero for negative *F*^2^. The threshold expression of *F*^2^ > σ(*F*^2^) is used only for calculating *R*-factors(gt) *etc*. and is not relevant to the choice of reflections for refinement. *R*-factors based on *F*^2^ are statistically about twice as large as those based on *F*, and *R*- factors based on ALL data will be even larger.

### Fractional atomic coordinates and isotropic or equivalent isotropic displacement parameters (Å^2^)

**Table:**

	*x*	*y*	*z*	*U*_iso_*/*U*_eq_	
Br1	0.32729 (8)	0.90633 (3)	0.26426 (13)	0.0702 (3)	
Br2	0.40752 (9)	1.03175 (3)	0.25156 (12)	0.0705 (3)	
Br3	0.73585 (10)	1.06551 (3)	0.23297 (14)	0.0807 (3)	
Br4	0.98266 (9)	0.97499 (3)	0.23443 (13)	0.0773 (3)	
N1	0.5436 (6)	0.7996 (2)	0.8034 (8)	0.0546 (19)	
H1A	0.5376	0.8133	0.8986	0.082*	
H1B	0.6186	0.8151	0.7820	0.082*	
H1C	0.5631	0.7656	0.8196	0.082*	
O1	0.8313 (6)	0.8333 (2)	0.1273 (8)	0.0672 (18)	
O2	0.9937 (7)	0.8565 (2)	0.3871 (9)	0.101 (3)	
O3	0.6431 (6)	0.8063 (2)	0.3863 (8)	0.076 (2)	
O4	0.4945 (6)	0.8125 (2)	0.1079 (8)	0.0713 (19)	
O5	0.7663 (7)	0.8481 (2)	0.7090 (7)	0.104 (2)	
H5	0.7413	0.8383	0.6081	0.157*	
C1	0.8783 (9)	0.8620 (3)	0.2667 (14)	0.050 (3)	
C2	0.5820 (10)	0.8306 (3)	0.2499 (14)	0.050 (3)	
C3	0.7630 (7)	0.9042 (3)	0.2521 (9)	0.0369 (19)	
C4	0.6200 (8)	0.8886 (3)	0.2517 (9)	0.040 (2)	
C5	0.5158 (7)	0.9276 (3)	0.2534 (9)	0.040 (2)	
C6	0.5515 (7)	0.9794 (3)	0.2527 (9)	0.047 (2)	
C7	0.6915 (8)	0.9947 (3)	0.2450 (10)	0.045 (2)	
C8	0.7960 (7)	0.9565 (3)	0.2469 (9)	0.040 (2)	
C9	0.9329 (9)	0.7886 (3)	0.1323 (11)	0.097 (3)	
H9A	1.0367	0.8006	0.1685	0.146*	
H9B	0.9010	0.7734	0.0185	0.146*	
H9C	0.9266	0.7629	0.2131	0.146*	
C10	0.3935 (9)	0.8076 (3)	0.6508 (10)	0.056 (2)	
H10A	0.3730	0.8447	0.6333	0.068*	
H10B	0.4018	0.7937	0.5466	0.068*	
C11	0.2643 (8)	0.7820 (3)	0.6773 (10)	0.081 (3)	
H11A	0.2763	0.7449	0.6766	0.121*	
H11B	0.1697	0.7919	0.5854	0.121*	
H11C	0.2630	0.7926	0.7872	0.121*	
C12	0.8988 (9)	0.8774 (3)	0.7572 (12)	0.104 (4)	
H12A	0.9141	0.8959	0.8627	0.155*	
H12B	0.8899	0.9019	0.6667	0.155*	
H12C	0.9844	0.8548	0.7757	0.155*	

### Atomic displacement parameters (Å^2^)

**Table:**

	*U*^11^	*U*^22^	*U*^33^	*U*^12^	*U*^13^	*U*^23^
Br1	0.0399 (5)	0.0808 (7)	0.1016 (9)	−0.0038 (5)	0.0403 (6)	0.0061 (6)
Br2	0.0615 (6)	0.0617 (6)	0.0956 (8)	0.0286 (5)	0.0386 (6)	0.0063 (6)
Br3	0.0898 (7)	0.0337 (5)	0.1331 (10)	−0.0058 (5)	0.0592 (7)	−0.0010 (6)
Br4	0.0455 (6)	0.0699 (7)	0.1306 (10)	−0.0122 (5)	0.0496 (6)	−0.0070 (6)
N1	0.061 (5)	0.042 (4)	0.072 (6)	−0.011 (3)	0.039 (4)	0.000 (4)
O1	0.057 (4)	0.063 (4)	0.077 (5)	0.027 (3)	0.022 (4)	−0.014 (4)
O2	0.068 (4)	0.114 (6)	0.085 (6)	0.048 (4)	−0.011 (4)	−0.033 (4)
O3	0.101 (5)	0.044 (4)	0.070 (5)	−0.006 (3)	0.020 (4)	0.019 (4)
O4	0.094 (5)	0.062 (4)	0.062 (5)	−0.038 (3)	0.035 (4)	−0.021 (4)
O5	0.101 (5)	0.150 (6)	0.074 (6)	−0.064 (4)	0.048 (4)	−0.018 (4)
C1	0.036 (6)	0.042 (6)	0.072 (9)	0.003 (5)	0.020 (6)	−0.009 (6)
C2	0.047 (6)	0.036 (6)	0.075 (9)	−0.006 (5)	0.035 (6)	−0.005 (6)
C3	0.031 (4)	0.028 (4)	0.057 (6)	−0.003 (4)	0.023 (4)	−0.005 (5)
C4	0.043 (5)	0.032 (5)	0.043 (6)	0.007 (4)	0.017 (5)	−0.002 (4)
C5	0.026 (4)	0.038 (5)	0.060 (6)	−0.007 (4)	0.021 (4)	−0.004 (5)
C6	0.035 (5)	0.045 (6)	0.058 (6)	0.014 (4)	0.016 (5)	−0.001 (4)
C7	0.032 (5)	0.033 (5)	0.068 (6)	−0.003 (4)	0.019 (5)	0.003 (4)
C8	0.016 (4)	0.041 (5)	0.062 (6)	−0.001 (4)	0.014 (4)	−0.002 (5)
C9	0.090 (7)	0.080 (7)	0.106 (9)	0.045 (6)	0.022 (6)	−0.025 (6)
C10	0.077 (6)	0.046 (6)	0.040 (6)	0.018 (5)	0.015 (6)	0.009 (5)
C11	0.052 (6)	0.097 (7)	0.085 (8)	−0.013 (5)	0.017 (6)	0.016 (6)
C12	0.082 (7)	0.100 (8)	0.144 (10)	−0.050 (6)	0.060 (7)	−0.030 (7)

### Geometric parameters (Å, °)

**Table:**

Br1—C5	1.902 (6)	C3—C4	1.411 (8)
Br2—C6	1.911 (6)	C4—C5	1.410 (8)
Br3—C7	1.876 (6)	C5—C6	1.370 (8)
Br4—C8	1.871 (6)	C6—C7	1.408 (7)
N1—C10	1.509 (8)	C7—C8	1.389 (8)
N1—H1A	0.8900	C9—H9A	0.9600
N1—H1B	0.8900	C9—H9B	0.9600
N1—H1C	0.8900	C9—H9C	0.9600
O1—C1	1.301 (9)	C10—C11	1.478 (8)
O1—C9	1.487 (7)	C10—H10A	0.9700
O2—C1	1.171 (9)	C10—H10B	0.9700
O3—C2	1.227 (9)	C11—H11A	0.9600
O4—C2	1.241 (9)	C11—H11B	0.9600
O5—C12	1.381 (7)	C11—H11C	0.9600
O5—H5	0.8200	C12—H12A	0.9600
C1—C3	1.507 (9)	C12—H12B	0.9600
C2—C4	1.530 (9)	C12—H12C	0.9600
C3—C8	1.381 (7)		
			
C10—N1—H1A	109.5	C6—C7—Br3	120.5 (5)
C10—N1—H1B	109.5	C3—C8—C7	121.2 (6)
H1A—N1—H1B	109.5	C3—C8—Br4	118.6 (5)
C10—N1—H1C	109.5	C7—C8—Br4	120.2 (5)
H1A—N1—H1C	109.5	O1—C9—H9A	109.5
H1B—N1—H1C	109.5	O1—C9—H9B	109.5
C1—O1—C9	114.7 (6)	H9A—C9—H9B	109.5
C12—O5—H5	109.5	O1—C9—H9C	109.5
O2—C1—O1	125.5 (8)	H9A—C9—H9C	109.5
O2—C1—C3	124.2 (9)	H9B—C9—H9C	109.5
O1—C1—C3	110.4 (7)	C11—C10—N1	112.1 (6)
O3—C2—O4	126.6 (8)	C11—C10—H10A	109.2
O3—C2—C4	117.4 (8)	N1—C10—H10A	109.2
O4—C2—C4	116.0 (8)	C11—C10—H10B	109.2
C8—C3—C4	120.3 (6)	N1—C10—H10B	109.2
C8—C3—C1	122.3 (6)	H10A—C10—H10B	107.9
C4—C3—C1	117.4 (6)	C10—C11—H11A	109.5
C5—C4—C3	118.2 (6)	C10—C11—H11B	109.5
C5—C4—C2	121.9 (7)	H11A—C11—H11B	109.5
C3—C4—C2	119.9 (6)	C10—C11—H11C	109.5
C6—C5—C4	121.0 (6)	H11A—C11—H11C	109.5
C6—C5—Br1	121.0 (5)	H11B—C11—H11C	109.5
C4—C5—Br1	118.0 (5)	O5—C12—H12A	109.5
C5—C6—C7	120.6 (6)	O5—C12—H12B	109.5
C5—C6—Br2	120.3 (5)	H12A—C12—H12B	109.5
C7—C6—Br2	119.0 (6)	O5—C12—H12C	109.5
C8—C7—C6	118.8 (6)	H12A—C12—H12C	109.5
C8—C7—Br3	120.8 (5)	H12B—C12—H12C	109.5
			
C9—O1—C1—O2	3.5 (13)	C2—C4—C5—Br1	2.8 (10)
C9—O1—C1—C3	−176.8 (6)	C4—C5—C6—C7	1.9 (11)
O2—C1—C3—C8	64.3 (12)	Br1—C5—C6—C7	179.9 (5)
O1—C1—C3—C8	−115.3 (8)	C4—C5—C6—Br2	179.1 (5)
O2—C1—C3—C4	−112.9 (10)	Br1—C5—C6—Br2	−2.9 (9)
O1—C1—C3—C4	67.5 (9)	C5—C6—C7—C8	−3.2 (11)
C8—C3—C4—C5	−2.7 (10)	Br2—C6—C7—C8	179.6 (6)
C1—C3—C4—C5	174.6 (7)	C5—C6—C7—Br3	177.0 (6)
C8—C3—C4—C2	177.5 (7)	Br2—C6—C7—Br3	−0.2 (8)
C1—C3—C4—C2	−5.3 (11)	C4—C3—C8—C7	1.4 (11)
O3—C2—C4—C5	−104.5 (9)	C1—C3—C8—C7	−175.7 (8)
O4—C2—C4—C5	77.2 (10)	C4—C3—C8—Br4	−176.4 (5)
O3—C2—C4—C3	75.3 (10)	C1—C3—C8—Br4	6.4 (11)
O4—C2—C4—C3	−103.0 (8)	C6—C7—C8—C3	1.5 (11)
C3—C4—C5—C6	1.0 (10)	Br3—C7—C8—C3	−178.7 (5)
C2—C4—C5—C6	−179.1 (7)	C6—C7—C8—Br4	179.3 (5)
C3—C4—C5—Br1	−177.0 (5)	Br3—C7—C8—Br4	−0.9 (9)

### Hydrogen-bond geometry (Å, °)

**Table:**

*D*—H···*A*	*D*—H	H···*A*	*D*···*A*	*D*—H···*A*
N1—H1A···O4^i^	0.89	1.94	2.772 (9)	155
N1—H1B···O5	0.89	1.92	2.810 (9)	174
N1—H1C···O3^ii^	0.89	1.99	2.871 (7)	170
N1—H1C···O4^ii^	0.89	2.58	3.249 (8)	132
O5—H5···O3	0.82	1.90	2.705 (8)	165
C10—H10A···Br3^iii^	0.97	2.92	3.736 (8)	143
C11—H11B···O2^iv^	0.96	2.47	3.357 (10)	153

Symmetry codes: (i) *x*, *y*, *z*+1; (ii) *x*, −*y*+3/2, *z*+1/2; (iii) −*x*+1, −*y*+2, −*z*+1; (iv) *x*−1, *y*, *z*.
